# Genetic insights into breast cancer risk

**DOI:** 10.1186/1897-4287-13-S1-A1

**Published:** 2015-09-09

**Authors:** Michelle Wong-Brown, Kelly Avery-Kiejda, Rodney J Scott

**Affiliations:** 1 Information Based Medicine Program, Hunter Medical Research Institute, NSW, Australia; 2 School of Biomedical Sciences, University of Newcastle, Newcastle, UK; 3 Division of Molecular Medicine, Hunter Area Pathology Service, Pathology North, NSW, Australia

## 

Since the identification of *BRCA1* and *BRCA2* much has been learned about the role of mutations in these genes and how they relate to disease risk. The primary population that has been screened for mutations in these genes has been women who have a family history of early onset breast and or ovarian cancer. The tumour characteristics of women who harbour a *BRCA1* mutation are similar to those identified in women who have been diagnosed with breast cancer that is estrogen, progesterone and EGFR (HER2) receptor negative, the so-called triple negative phenotype (TNBC). Screening of women with a family history that is consistent with that found in women who have a *BRCA1* mutation at best yields a 20% identification rate, leaving approximately 80% of women without a diagnosis of disease. Much effort has been expended in searching for additional breast cancer susceptibility genes, which has met with limited success but the overall numbers of women with mutations in these additional genes is low and implementation of this information into clinical practice is proving difficult.

In an effort to identify additional women who may benefit from knowledge about their *BRCA1* and *BRCA2* status we undertook a study, using next generation DNA sequencing, examining a series of unselected TNBC patients for mutations in *BRCA1* and *BRCA2*. In parallel, we examined the recently identified *PALB2* in the same series to women to determine how many could be accounted for by this gene.

Since a large proportion of women with a family history of disease display an identical family and tumour phenotype to women with *BRCA1* or *BRCA2* mutation we considered it not unlikely that many of them possibly harbour mutations in these two genes in regions that are not typically screened for using current technologies. The results of a pilot study will be presented that suggest re-sequencing of *BRCA1* and *BRCA2*, in their entirety, is warranted.

In summary, there is much to be gained by re-sequencing BRCA1 and BRCA2 especially given that the coding sequence represents less than 10% of each gene and the source of patients should be expanded to TNBC patients, to capture those women who do not have any obvious family history.

